# Dataset for amiodarone adverse events compared to placebo using data from randomized controlled trials

**DOI:** 10.1016/j.dib.2019.104835

**Published:** 2019-11-18

**Authors:** Morgan K. Moroi, Mohammed Ruzieh, Nader M. Aboujamous, Mehrdad Ghahramani, Gerald V. Naccarelli, John Mandrola, Andrew J. Foy

**Affiliations:** a Penn State College of Medicine, Hershey, PA, USA; b Penn State Heart and Vascular Institute, Hershey, PA, USA; c Penn State Department of Internal Medicine, Hershey, PA, USA; d Baptist Health Louisville, Louisville, KY, USA

**Keywords:** Amiodarone, Adverse events, Toxicity

## Abstract

The dataset presented here provides a detailed description of the adverse events of amiodarone versus placebo using data from 43 randomized controlled trials. Two authors (M.M., M.R.) independently extracted the data. The dataset also includes baseline patient characteristics, amiodarone loading and maintenance doses, as well as forest plots describing the relative risk (RR) of developing an adverse event related to the pulmonary, thyroid, hepatic, cardiac, skin, gastrointestinal, neurological, and ocular systems. The Mantel-Haenszel random effects model was used to determine the relative risk of adverse events of amiodarone compared to placebo. This dataset is complementary to our article “Meta-analysis Comparing the Relative Risk of Adverse Events for Amiodarone Versus Placebo”, which was published in the American Journal of Cardiology [1]. The data can be used to assess certain adverse events and their relation to amiodarone loading and/or maintenance dose.

**Table:** Specifications Table

Subject	Cardiology and Cardiovascular Medicine
Specific subject area	A meta-analysis reporting the relative risk of developing adverse events related to amiodarone compared to placebo
Type of data	Tables Figures Raw data (supplement)
How data were acquired	We searched PubMed, Google Scholar, the Cochrane Central Register for RCTs, and ClinicalTrials.gov for studies that evaluated amiodarone use irrespective of indication or efficacy of amiodarone therapy
Data format	Raw, Analyzed, Filtered
Parameters for data collection	Patients who took amiodarone for prevention and/or treatment of ventricular or atrial arrhythmias.
Description of data collection	We searched PubMed, Google Scholar, the Cochrane Central Register for RCTs, and ClinicalTrials.gov for studies that evaluated amiodarone use irrespective of indication or efficacy of amiodarone therapy. Key search terms used were amiodarone, adverse events, side effects, placebo, atrial fibrillation, atrial flutter, ventricular tachycardia, arrhythmias, liver, hepatic, skin, thyroid, eye, and lung, and pulmonary. Bibliographies of retrieved studies were hand-searched to identify additional relevant studies.
Data source location	Data from randomized controlled trials.
Data accessibility	With the article, and the supplement.
Related research article	Ruzieh M, Moroi MK, Aboujamous NM, Ghahramani M, Naccarelli GV, Mandrola J, Foy AJ. Meta-Analysis Comparing the Relative Risk of Adverse Events for Amiodarone Versus Placebo. Am J Cardiol. 2019. pii: S0002-9149(19)31046-X. https://doi.org/10.1016/j.amjcard.2019.09.008. [Epub ahead of print]

**Table:** 

**Value of the Data** • This dataset provides detailed description of the adverse events and its relative risk in patients taking amiodarone compared to placebo. This is very important for the medical community as amiodarone is one of commonly used drugs to treat atrial fibrillation. • Medical providers who are prescribing or managing patients taking amiodarone as well as researchers interested in assessing amiodarone related adverse events. • Further analysis could be performed to determine how different amiodarone loading and maintenance regimens could affect the development of amiodarone related adverse events. • Understanding the nature and the rate of amiodarone related adverse events will help physicians develop appropriate screening and monitoring strategies for these events.

## 1 Data

**Fig. 1:**
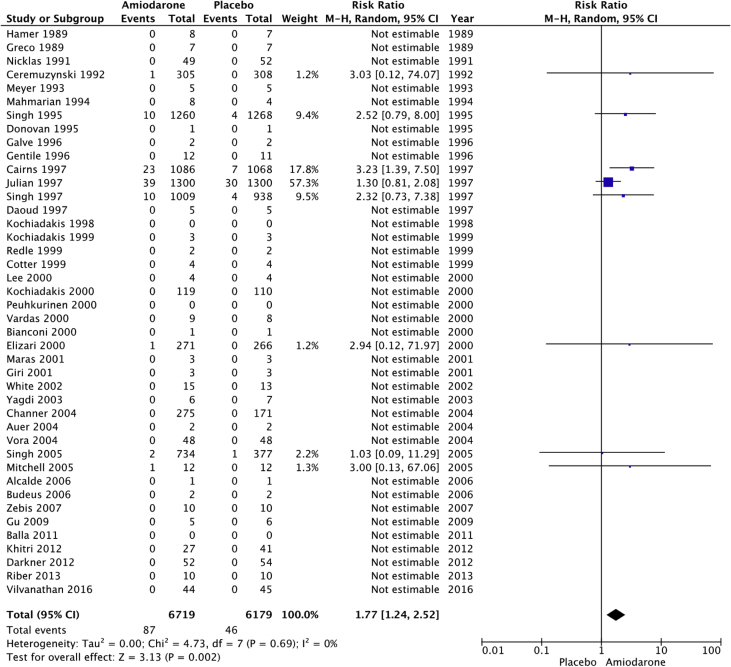
Pulmonary adverse events. “Total” represents total events per 10,000 person-years. The incident rate of pulmonary adverse events per 10,000 person-years was higher in the amiodarone group versus placebo (129 vs 74; RR: 1.77; 95% CI [1.24–2.52], P = 0.002, 1^2^: 0%).

**Fig. 2:**
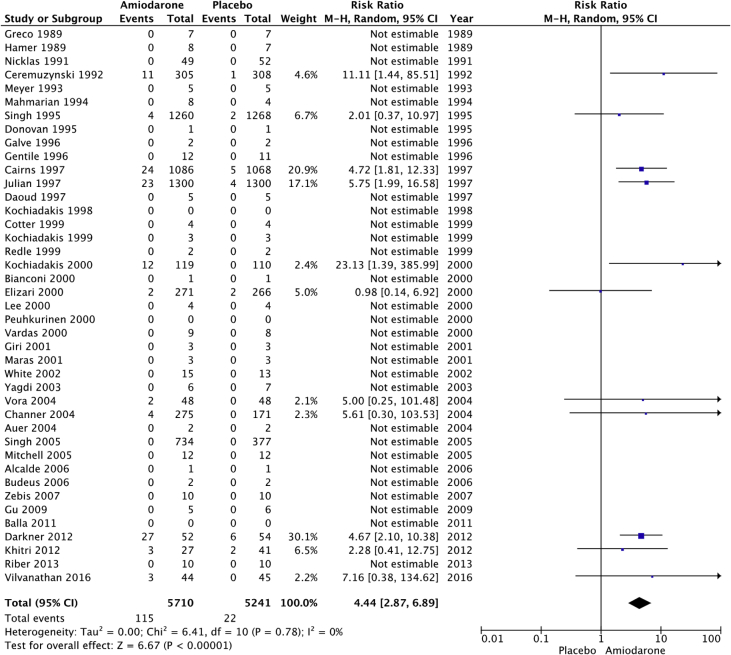
Thyroid adverse events. “Total” represents total events per 10,000 person-years. The incident rate of thyroid adverse events per 10,000 person-years was higher in the amiodarone group versus placebo (201 vs 42; RR: 4.44; 95% CI [2.87–6.89], P < 0.001, 1^2^: 0%).

**Fig. 3:**
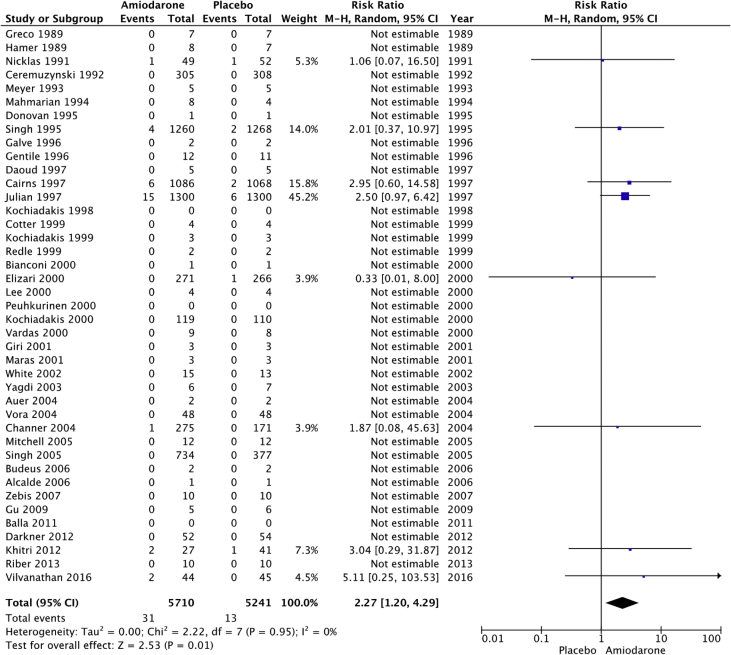
Liver adverse events. “Total represents total events per 10,000 person-years. Liver adverse events were rare, but the rate of liver adverse events per 10,000 person-years was still higher in the amiodarone group versus placebo (54 vs 25; RR: 2.27; 95% CI [1.20–4.29], P = 0.01, I^2^: 0%).

**Fig. 4:**
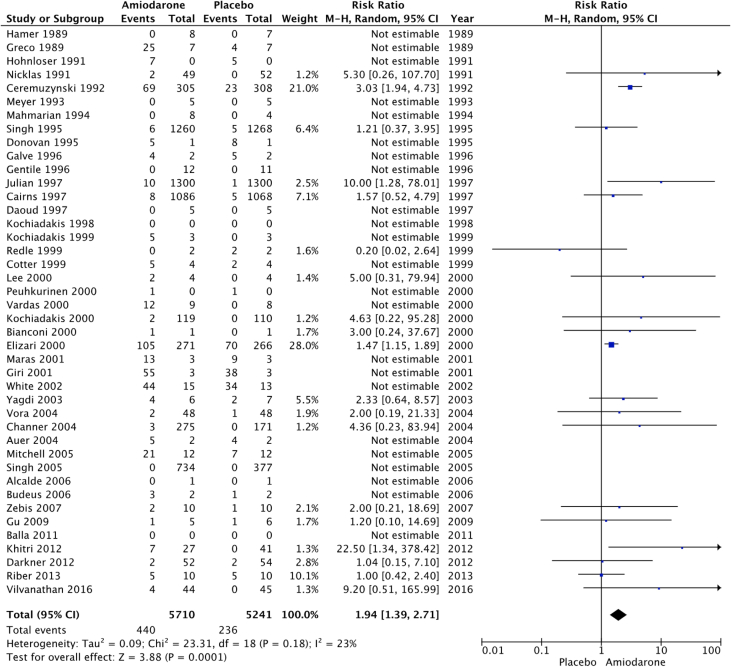
Cardiac adverse events. “Total” represents total events per 10,000 person-years. Cardiac adverse events were the most commonly reported adverse events for both groups. The incident rate of cardiac adverse events per 10,000 person-years was higher in patients receiving amiodarone versus placebo (771 vs 450; RR: 1.94; 95% CI [1.39–2.71], P = 0.0001, I^2^: 23%).

**Fig. 5:**
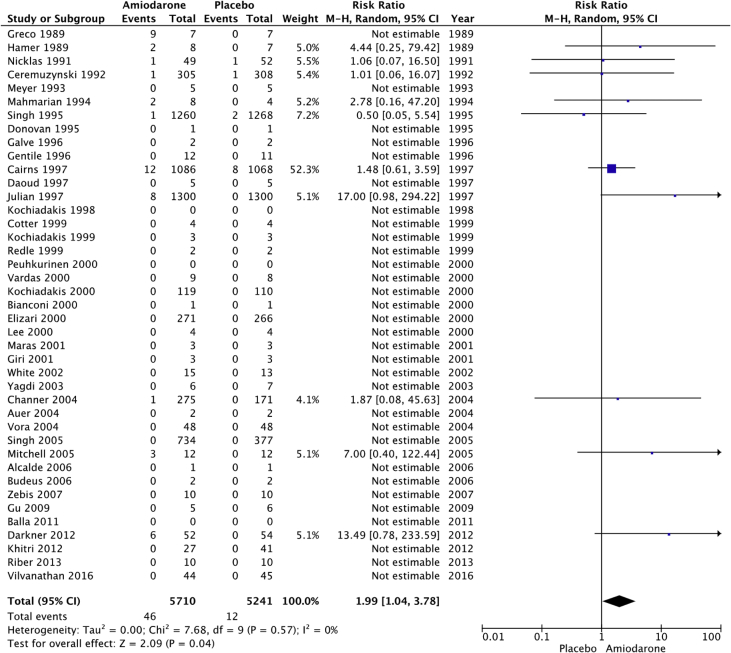
Skin adverse events. “Total” represents total events per 10,000 person-years. The incident rate of skin adverse events was higher in the amiodarone group versus placebo (81 vs 23; RR: 1.99; 95% CI [1.04–3.78], P = 0.04, I^2^: 0%).

**Fig. 6:**
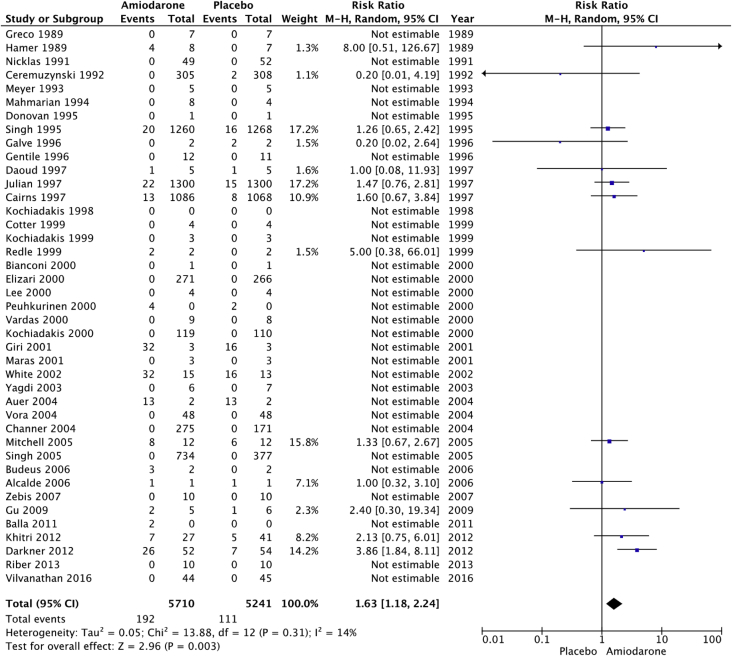
Gastrointestinal adverse events. “Total” represents total events per 10,000 person-years. The incident rate of gastrointestinal adverse events was higher in patients receiving amiodarone compared to those receiving placebo (336 vs 212; RR: 1.63; 95% CI [1.18–2.24], P = 0.003, I^2^: 14%).

**Fig. 7:**
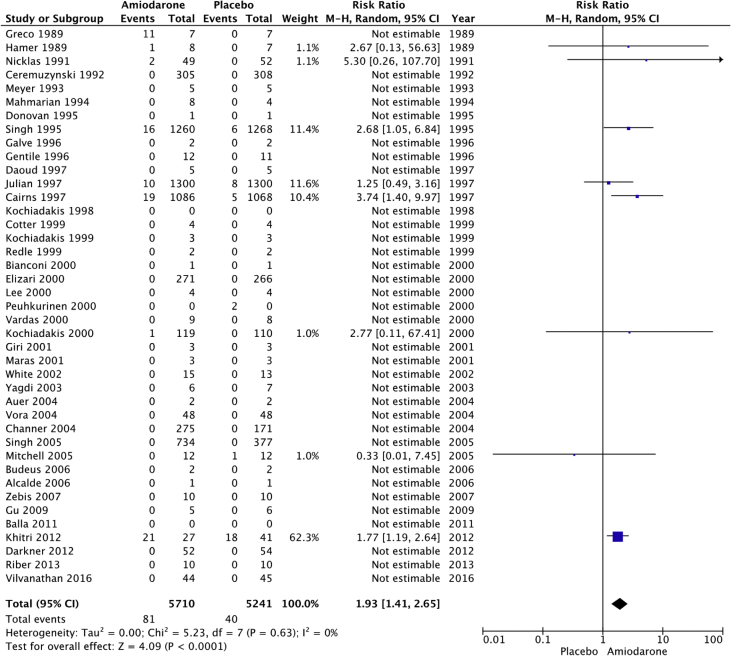
Neurological adverse events. “Total” represents total events per 10,000 person-years. The incident rate of neurological adverse events per 10,000 person-years was higher in the amiodarone group versus placebo (140 vs 76; RR: 1.93; 95% CI [1.41–2.65], P < 0.001, 1^2^: 0%).

**Fig. 8:**
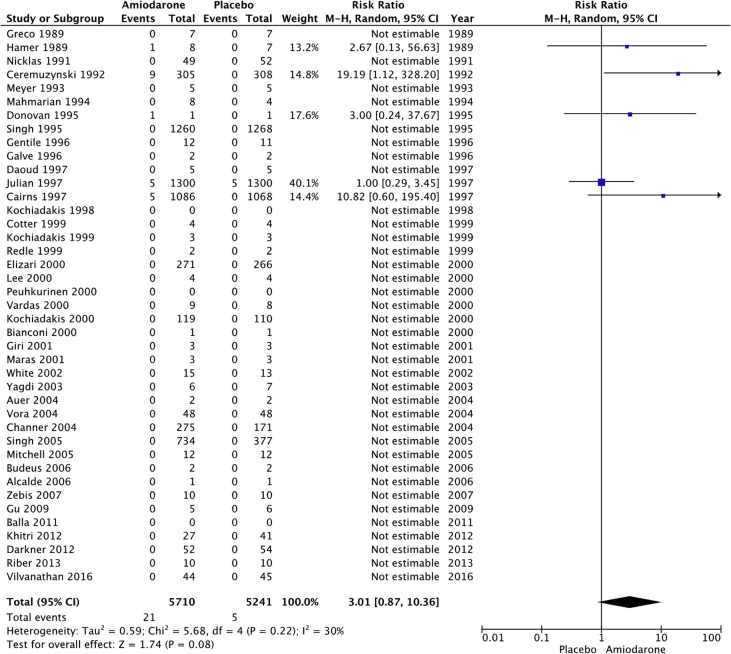
Ocular adverse events. “Total” represents total events per 10,000 person-years. The incident rate of ocular adverse events per 10,000 person-years was higher in patients receiving amiodarone versus placebo; however, this never reached statistical significance (37 vs 10; RR: 3.01; 95% CI [0.87–10.36], P = 0.08, I^2^: 30%).

**Fig. 9:**
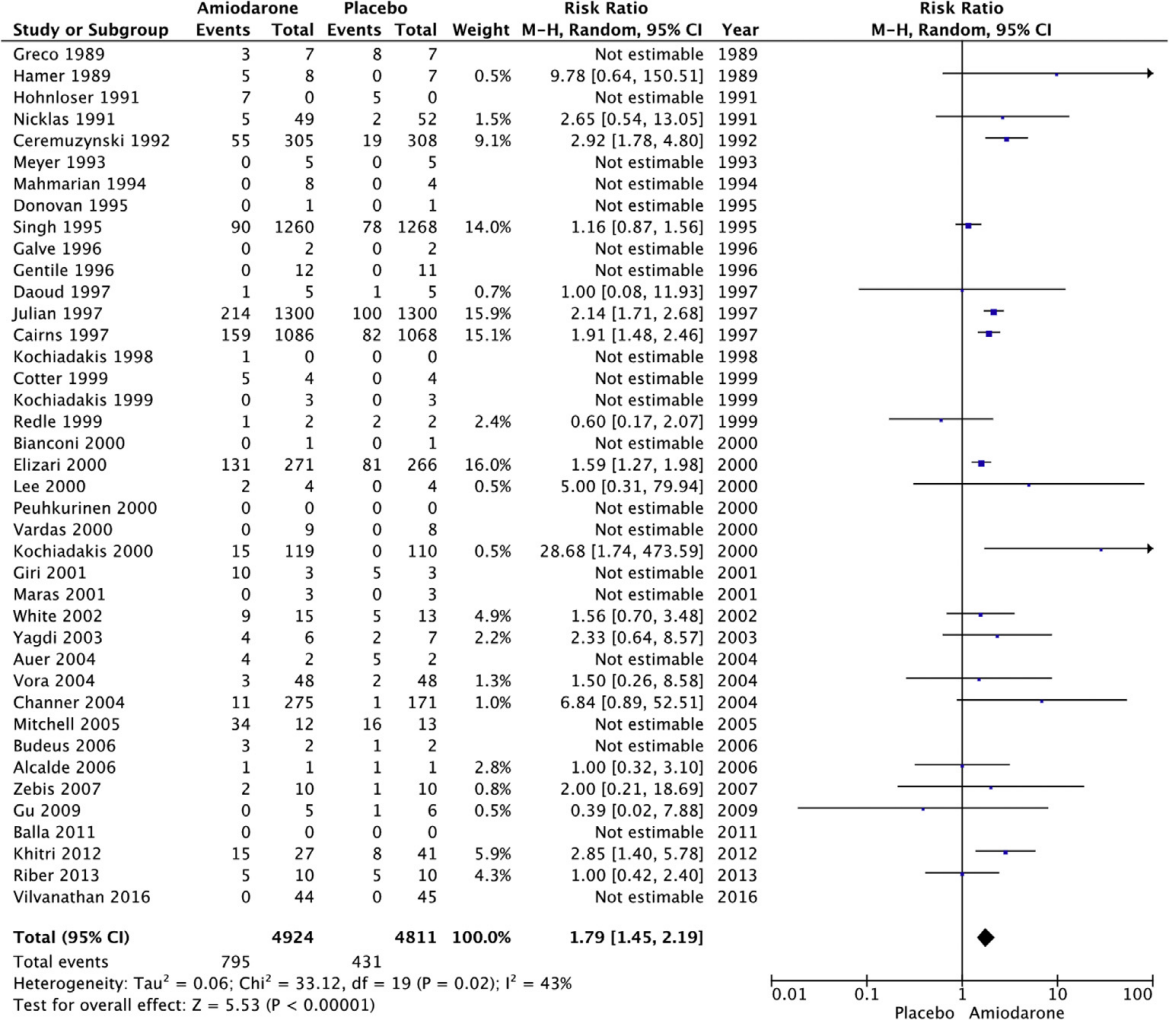
Rates of drug discontinuation. “Total” represents total events per 10,000 person-years. The incident rate of drug discontinuation secondary to side effects per 10,000 person-years was higher in the amiodarone group versus placebo (1614 vs 896; RR: 1.79; 95% CI [1.45–2.19], P < 0.001, I^2^: 43%).

The raw dataset contains the number of events and number of patient-year for the amiodarone and placebo arm of each study (reads in xlsx format, each organ system in a separate sheet). Patients’ characteristics are summarized in Table 1, Table 2. The number and incident rate of events are listed in Table 4. The rate of adverse events in the amiodarone arm for each organ system, and the rate of drug discontinuation compared to placebo are illustrated in Fig. 1, Fig. 2, Fig. 3, Fig. 4, Fig. 5, Fig. 6, Fig. 7, Fig. 8, Fig. 9.

**Table 1:** Baseline patient characteristics. Forty-three randomized control trials [[2], [3], [4], [5], [6], [7], [8], [9], [10], [11], [12], [13], [14], [15], [16], [17], [18], [19], [20]] were studied, and 11,395 patients were included (5792 patients in the amiodarone group, 5603 patients in the placebo group). Average age was 62.0 years for patients receiving amiodarone and 62.3 years for patients receiving placebo. Follow up time ranged from 1 week–6 months for studies with follow up < 12 months. Indications for amiodarone therapy were suppression of atrial and ventricular arrhythmias, and maintenance dose for amiodarone ranged from 200 to 600 mg daily. Raw data for the adverse events is provided in the supplement material.

											Amiodarone arm			Placebo arm		
First author	Year	Medical condition	Average Ejection fraction	Percent with IHD	Reason for intervention	Mean follow-up (days)	Average Load Dose (mg/day)	Load (# of days)	Average Maintenance Dose (mg/day)	Maintenance (# of days)	No. Of Pts	Mean age (yrs)	Male Gender (%)	No. Of Pts	Mean age (yrs)	Male Gender (%)
Greco	1989	Patients with anterior MI	NA	100%	Reduce mortality and morbidity	Until discharge	10–20 mg/kg	1	N/A	N/A	159	54	85	160	55	87
Hamer	1989	Congestive heart failure	18%	60%	Arrhythmia control, exercise tolerance and ventricular function	180	387	180	200	150	16	70	N/A	14	66	N/A
Hohnloser	1991	Post CABG	NA	100%	Suppression of SVT and ventricular arrhythmias	4	1125	4	N/A	N/A	39	59	76.9	38	59	73.7
Meyer	1993	Stable angina	59%	100%	Limiting angina pectoris	60	400	30	200	50	32	61	N/A	31	58	N/A
Mahmarian	1994	Systolic heart failure and NSVT	24%	49%	Suppression of ventricular arrhythmias	90	422	30	50 or 100	54	32	53.5	77.5	16	51	81
Donovan	1995	Patients with recent-onset AF	NA	48%	Restoration of sinus rhythm	Until discharge	7 mg/kg	1	N/A	N/A	32	56	N/A	32	59	N/A
Galve	1996	Newly diagnosed AF	NA	NA	Rhythm control	15	1200 + 5 mg/kg	1	N/A	N/A	50	60	54	50	61	56
Gentile	1996	Elderly patients with systolic heart failure	<40%	61%	Reduce sudden cardiac death	180	400	30	100	150	24	71	N/A	22	71	N/A
Daoud	1997	Patients undergoing open heart surgery	48%	60%	Prevention of post-op AF	30	200–1000	13 ± 7	N/A	N/A	64	57	68.8	60	67	66.7
Kochiadakis	1998	Patients with recent onset AF	50%	NA	Restoration of sinus rhythm	1	2100 + 20 mg/kg	1	N/A	N/A	48	63	56	49	65	51
Cotter	1999	Patients with paroxysmal AF	Majority <45%	43%	Restoration of sinus rhythm	30	3000	1	N/A	N/A	50	64.5	48	50	68	38
Kochiadakis	1999	Patients with persistent AF	50%	NA	Restoration of sinus rhythm	30	460 + 20 mg/kg	28	N/A	N/A	33	64	48.5	34	63	47.1
Redle	1999	Patients undergoing CABG	49%	100%	Prevention of post-op AF	10	430	11	N/A	N/A	73	63	83.5	70	64.5	81.4
Bianconi	2000	Patients with AF or AFL	NA	15%	Acute termination of AF or flutter	3–7	5 mg/kg	1	N/A	N/A	54	63	57	54	66	54
Elizari	2000	Patients with acute MI	NA	100%	Reduce morbidity/mortality	180	900	3	N/A	N/A	542	60.3	80.6	531	60.5	75.1
Lee	2000	Patients undergoing CABG	59%	100%	Prevention of post-op AF	18	150 + 0.4/kg	8	N/A	N/A	74	66	54	76	65	55
Peuhkurinen	2000	Patients with recent-onset AF	63%	21%	Restoration of sinus rhythm	1	30 mg/kg	1	N/A	N/A	31	56	81	31	62	65
Vardas	2000	Patients with AF	51%	NA	Restoration of sinus rhythm	30	600	28	N/A	N/A	108	64	49.1	100	65	49
Giri	2001	Patients undergoing CABG, valve or combined	43%	98%	Prevention of post-op AF	9	1000	6; 10	N/A	N/A	120	72.7	78	100	72.5	74
Maras	2001	Patients undergoing CABG	44%	100%	Prevention of post-op AF	7	325	8	N/A	N/A	159	58.3	80	156	57.3	76
White	2002	Patients undergoing open heart surgery	43%	35%	Prevention of post-op AF	21–42	1200–1400	>10; >6	N/A	N/A	120	72.6	78.3	100	72.5	74
Yagdi	2003	Patients undergoing CABG	48%	100%	Prevention of post-op AF	30	400-600 + 10/kg	2; 5; 5	N/A	N/A	77	59.3	80.5	80	61.1	73.7
Auer	2004	Patients undergoing open heart surgery	69%	64%	Prevention of post-op AF	12	667	9	N/A	N/A	63	64	58.7	65	63	58.5
Mitchell	2005	Patients undergoing CABG, valve replacement, repair	58%	75%	Prevention of post-op atrial tachyarrhythmia	13	10 mg/kg	13	N/A	N/A	299	61.3	82.6	302	61.9	81.8
Alcalde	2006	Patients undergoing CABG	53%	100%	Prevention of post-op AF & AFL	10	1800	1–3	N/A	N/A	46	61	63	47	61.1	70.2
Budeus	2006	Patients undergoing CABG	63%	100%	Prevention of post-op AF	0.5	640	7	N/A	N/A	55	64.9	87.3	55	66.7	76.4
Zebis	2007	Patients undergoing CABG	55%	100%	Prevention of post-op AF	30	1200	5	N/A	N/A	125	67	86	125	67	80
Gu	2009	Patients undergoing off-pump CABG	61%	100%	Prevention of post-op AF	21	200 + 70 mg/kg	17	N/A	N/A	100	73.6	75	110	74.2	72
Balla	2011	Newly diagnosed AF	NA	NA	Rhythm control for AF	1	30 mg/kg	1	N/A	N/A	40	58.9	72.5	40	58.6	60
Khitri	2012	AF, AFL	59%	15%	Rhythm control	90	330	30	200	60	108	64.9	73.1	162	62.4	64.9
Riber	2013	Lung cancer surgery	NA	2%	Prevention of post-op AF	30	1200	5	N/A	N/A	122	66	49	120	67	47
Darkner	2014	AF patients undergoing RFA	50%	7%	Rhythm control after ablation	180	400	30	200	26	104	62	81	108	61	86

**AF**: Atrial fibrillation, **AFL:** Atrial flutter, **CABG:** Coronary artery bypass graft, **IHD:** Ischemic heart disease, **MI:** myocardial infarction, **NA:** Not available, **NSVT:** Non-sustained ventricular tachycardia, **RFA:** Radiofrequency ablation.

**Table 2:** Baseline patient characteristics. Forty-three randomized control trials [[2], [3], [4], [5], [6], [7], [8], [9], [10], [11], [12], [13], [14], [15], [16], [17], [18], [19], [20]] were studied, and 11,395 patients were included (5792 patients in the amiodarone group, 5603 patients in the placebo group). Average age was 62.0 years for patients receiving amiodarone and 62.3 years for patients receiving placebo. Follow up time ranged from 12–54 months in studies with follow up ≥ 12 months. Indications for amiodarone therapy were suppression of atrial and ventricular arrhythmias, and maintenance dose for amiodarone ranged from 200 to 600 mg daily. Raw data for the adverse events is provided in the supplement material.

											Amiodarone arm			Placebo arm		
First author	Year	Medical condition	Average ejection fraction	Percent with IHD	Reason for intervention	Mean follow-up (months)	Average Load dose (mg/day)	Average Load (day)	Average maintenance dose (mg)	Average maintenance (days)	No. of Pts	Mean age (year)	Male Gender (%)	No. of Pts	Mean age (year)	Male Gender (%)
Nicklas	1991	Heart failure and frequent ventricular ectopy	20%	52%	Reduce sudden cardiac death	12	400	28	200	215	49	56	83.7	52	59	86.5
Ceremuzynski	1992	Post MI	Majority > 40%	100%	Reduce mortality and ventricular arrhythmias	12	800	7	200–400	306	305	59.4	71.1	308	58.6	68.2
Singh[36]	1995	Patients with CHF and vent arrhythmia	<40%	71%	Improve mortality	45	800	14	328	1246	336	65	99.1	338	66.1	98.8
Cairns	1997	Survivors of MI with frequent or repetitive PVCs	NA	100%	Resuscitated ventricular fibrillation or arrhythmic death	21.5	20/kg	14	200–400	365–730	606	64	82.5	596	64	82
Julian	1997	Survivors of MI and EF ≤ 40%	30%	35%	All-cause mortality	21	450	112	200	253–618	743	59.6	83.8	743	60.2	84.9
Singh	1997	Patients with CHF, COPD and patients undergoing surgery	25–30%	NA	Evaluate pulmonary toxicity	45	800	14	300–400	365–1620	269	65	N/A	250	65.8	N/A
Kochiadakis	2000	Paroxysmal AF	55%	NA	Rhythm control	22	12.5/kg	14	200	720	65	63.2	52.3	60	62.8	51.7
Channer	2004	Persistent AF undergoing DCCV	59%	30%	Rhythm control	54	800	14	200	364	61	66	77	38	68	79
Vora	2004	Patients with chronic rheumatic AF	56%	NA	Rhythm or rate control	12	600	10	200	355	48	39.5	47.9	48	38	45.8
Singh	2005	Persistent AF	50%	25%	Rhythm control	12–54	700	28	200–300	>365	267	67.1	99.3	137	67.7	99.3
Vilvanathan	2016	AF in patients post BMV	58%	1%	Rhythm control for AF	12	500	28	200	365	44	38.8	20.5	45	37.62	34.1

**AF**: Atrial fibrillation, **BMV:** balloon mitral valvuloplasty, **CHF:** congestive heart failure, **COPD:** chronic obstructive pulmonary disease, **DCCV:** direct current cardioversion, **EF:** Ejection fraction, **IHD:** Ischemic heart disease, **MI:** myocardial infarction, **NA:** Not available, **PVC:** premature ventricular contraction.

## 2 Experimental design, materials, and methods

The protocol was developed by three authors (M.M., M.R., A.F.) and revised by all authors.

PubMed, Google Scholar, the Cochrane Central Register for randomized controlled trials, and ClinicalTrials.gov were searched for studies that analyzed the use of amiodarone regardless of indication or efficacy of therapy (latest search was conducted on October 10, 2018). Articles were identified using key search terms: amiodarone, adverse events, side effects, placebo, atrial fibrillation, atrial flutter, ventricular tachycardia, arrhythmias, liver, skin, thyroid, eye, and lung. References of all identified studies were also hand-searched for inclusion to identify additional relevant studies [1].

All articles were then independently reviewed for inclusion in this analysis by two authors (M.M., M.R.). Inclusion criteria were: 1) randomized control trial, 2) documentation of adverse events and drug discontinuation due to adverse events, 3) presence of placebo arm. Data on sample size, follow up, and outcomes were then extracted. Discrepancies were discussed and resolved by consensus.

Primary outcomes of this analysis were pulmonary, hepatic, thyroid, ocular, cardiac, skin, and neurological adverse events, as well as drug discontinuation related to adverse side effects. Specific adverse events within each organ system were also reported. All adverse events were presented as incident rate per 10,000 person-years.

The Cochrane Risk of Bias table and the Grading of Recommendations, Assessment, Development and Evaluation (GRADE) System were utilized to determine risk of bias and quality of the outcomes in all trials incorporated into this analysis (Table 3).

**Table 3:**
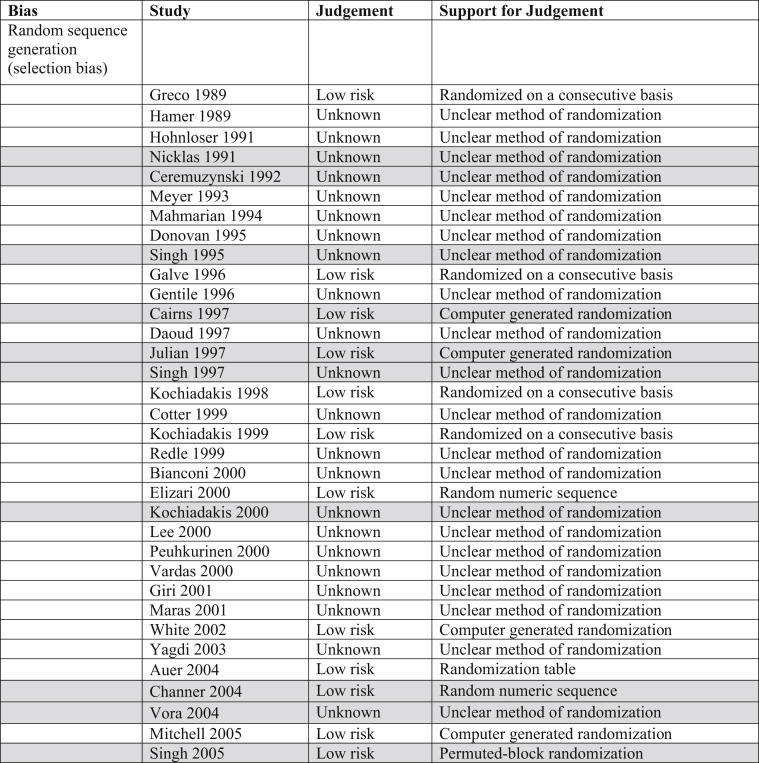

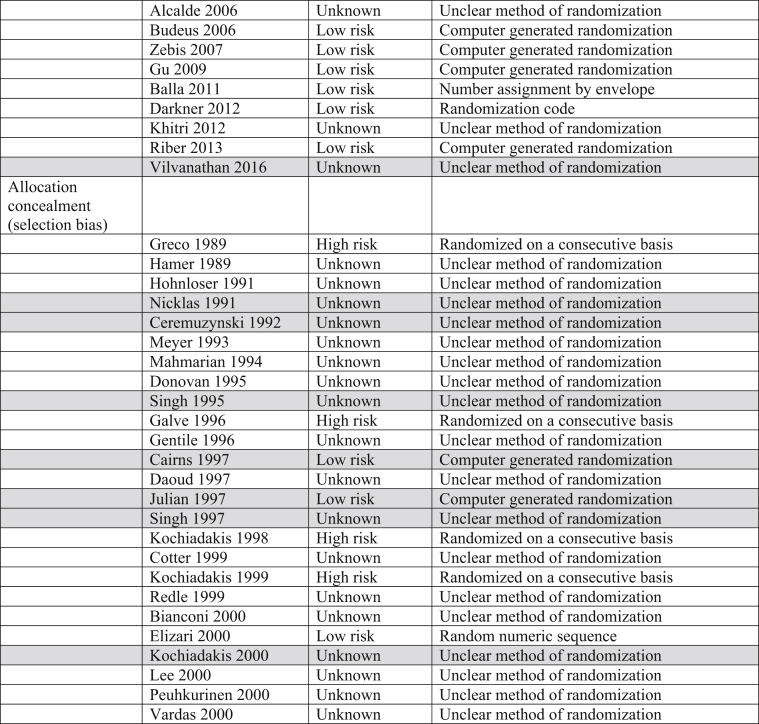

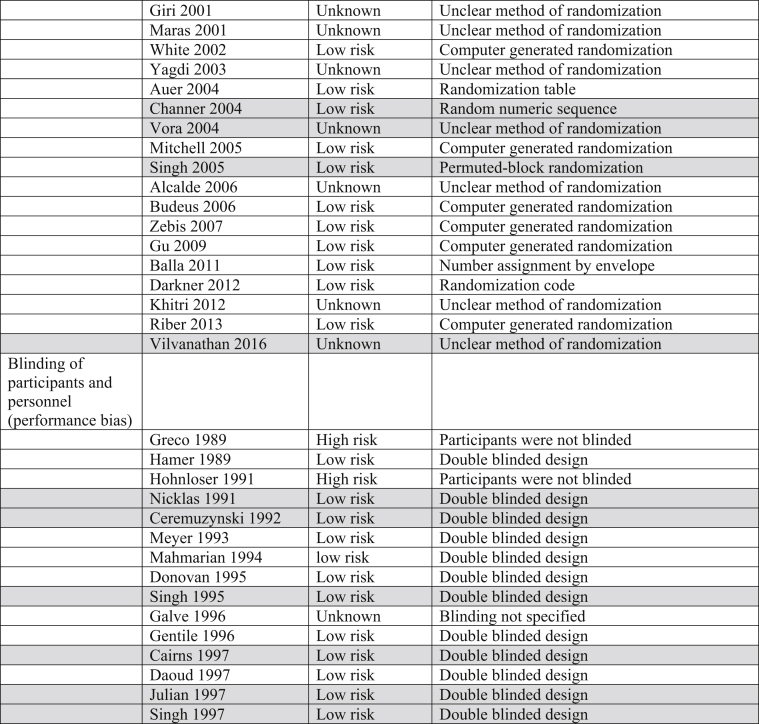

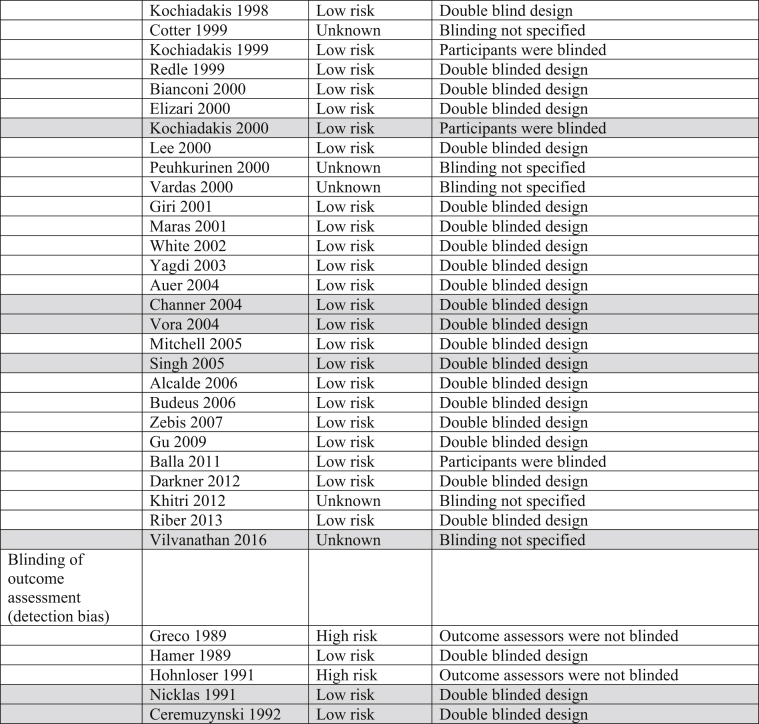

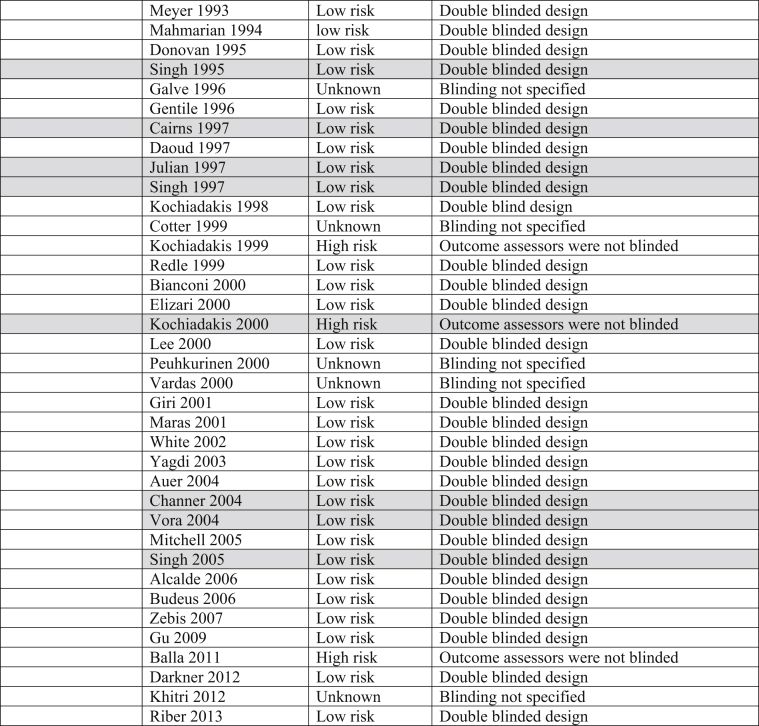

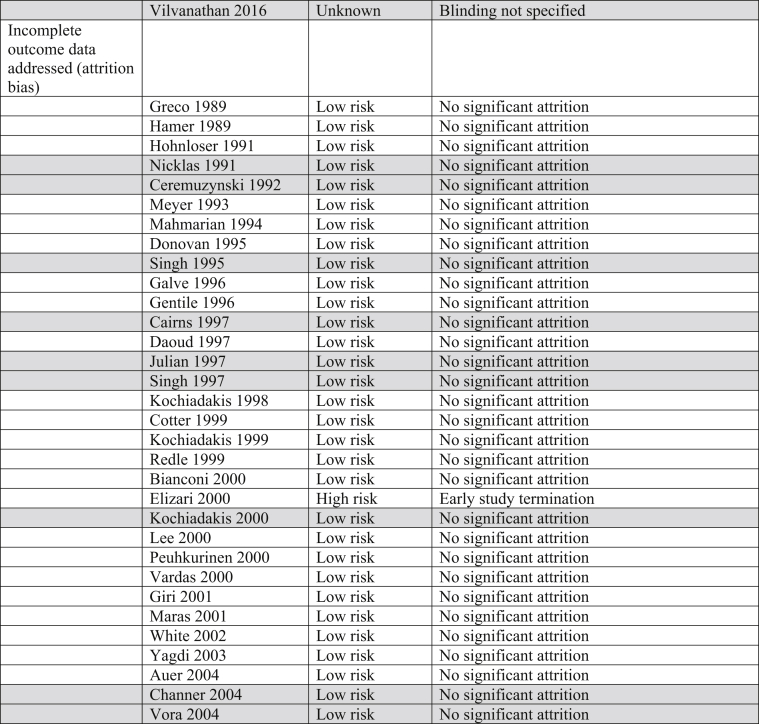

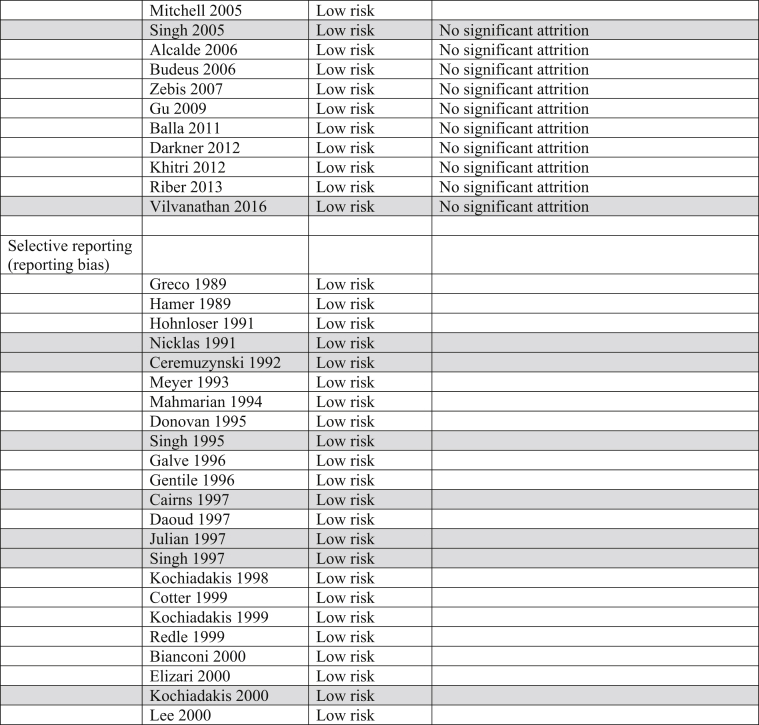

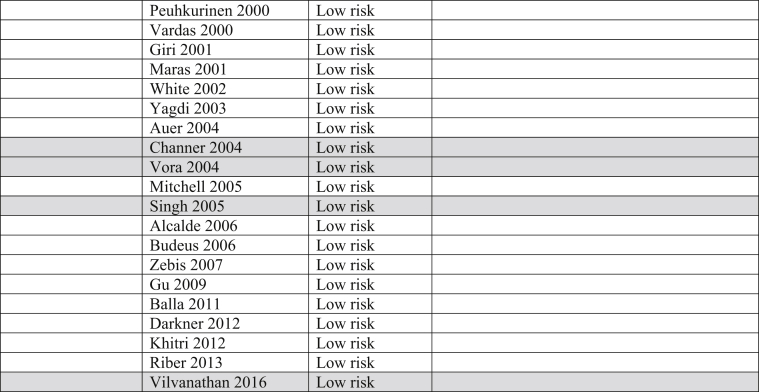
Risk of bias. Majority of trials included in this analysis were double blinded, decreasing both performance and detection biases.

Highlighted are studies with follow up ≥ 12 months.

**Table 4:** Number of events, incident rate, and relative risk of specific adverse events for amiodarone compared to placebo.

organ system			Follow up ≥ 12 months, No. of events (events/10,000 patient year)			All, No. of events (events/10,000 patient year)		
			Amiodarone arm	Placebo	RR (95% CI), P value	Amiodarone arm	Placebo	RR (95% CI), P value
Pulmonary adverse events		Pulmonary fibrosis	8 (13)	6 (11)		8 (12)	6 (11)	
		Cough	0 (0)	0 (0)		1 (1)	0 (0)	
		Lung infiltrates	0 (0)	0 (0)		1 (1)	0 (0)	
		Unspecified	77 (124)	40 (70)		77 (115)	40 (65)	
		**Total**	**85 (136)**	**46 (81)**	**1.74 (1.21**–**2.50), 0.003**	**87 (129)**	**46 (74)**	**1.77 (1.24**–**2.52), 0.002**
Thyroid adverse events		Clinical hyperthyroidism	19 (36)	4 (8)		19 (33)	5 (9)	
		Clinical hypothyroidism	27 (52)	0 (0)		27 (47)	0 (0)	
		Subclinical change in TFT	13 (25)	3 (6)		40 (70)	8 (15)	
		Unspecified	24 (46)	5 (11)		29 (51)	9 (17)	
		**Total**	**83 (159)**	**12 (25)**	**5.32 (2.99**–**9.44),** < **0.001**	**115 (201)**	**22 (42)**	**4.44 (2.87**–**6.89),** < **0.001**
Liver adverse events		Liver failure	0 (0)	0 (0)		0 (0)	0 (0)	
		Elevated liver enzymes	8 (15)	3 (6)		10 (18)	5 (10)	
		Unspecified	21 (40)	8 (17)		21 (37)	8 (15)	
		**Total**	**29 (56)**	**11 (23)**	**2.42 (1.23**–**4.74), 0.01**	**31 (54)**	**13 (25)**	**2.27 (1.20**–**4.29), 0.01**
Cardiac adverse events		Bradyarrhythmias	100 (192)	34 (72)		267 (468)	128 (244)	
		Hypotension	0 (0)	0 (0)		98 (172)	65 (124)	
		Long QT	5 (10)	0 (0)		18 (32)	0 (0)	
		Torsade de pointes	0 (0)	0 (0)		0 (0)	0 (0)	
		Worsening heart failure	1 (2)	1 (2)		5 (9)	5 (10)	
		Unspecified conduction disease	0 (0)	0 (0)		46 (81)	32 (61)	
		Unspecified	0 (0)	0 (0)		6 (11)	6 (11)	
		**Total**	**106 (203)**	**35 (74)**	**2.76 (1.91**–**3.98),** < **0.001**	**440 (771)**	**236 (450)**	**1.94 (1.39**–**2.71)** < **0.001**
Skin adverse events		Blue/gray discoloration of skin	2 (4)	3 (6)		2 (4)	3 (6)	
		Photosensitivity	1 (2)	0 (0)		11 (19)	0 (0)	
		Unspecified rash/flushing	21 (40)	9 (19)		33 (58)	9 (17)	
		**Total**	**24 (46)**	**12 (25)**	**1.51 (0.73**–**3.11), 0.26**	**46 (81)**	**12 (23)**	**1.99 (1.04**–**3.78), 0.04**
GI adverse events		Dyspepsia/nausea/vomiting	20 (38)	16 (34)		122 (214)	74 (141)	
		Diarrhea	0 (0)	0 (0)		8 (14)	4 (8)	
		Unspecified	35 (67)	25 (53)		62 (109)	33 (63)	
		**Total**	**55 (105)**	**41 (86)**	**1.36 (0.91**–**2.04), 0.14**	**192 (336)**	**111 (212)**	**1.63 (1.18**–**2.24), 0.003**
Neuro adverse events		Ataxia or gait disturbances	17 (33)	6 (13)		17 (30)	6 (11)	
		Headache	0 (0)	0 (0)		25 (44)	17 (32)	
		Dizziness	0 (0)	0 (0)		7 (12)	4 (8)	
		Tremor	2 (4)	0 (0)		2 (4)	0 (0)	
		Peripheral neuropathy	0 (0)	0 (0)		1 (2)	0 (0)	
		Unspecified	29 (56)	13 (27)		29 (51)	13 (25)	
		**Total**	**48 (92)**	**19 (40)**	**2.35 (1.38**–**4.00), 0.002**	**81 (140)**	**40 (76)**	**1.93 (1.41**–**2.65),** < **0.001**
Ocular adverse events		Corneal microdeposits	9 (17)	0 (0)		9 (16)	0 (0)	
		Blurred vision	0 (0)	0 (0)		1 (2)	0 (0)	
		Blue vision spots	0 (0)	0 (0)		1 (2)	0 (0)	
		Unspecified	10 (19)	5 (11)		10 (18)	5 (10)	
		**Total**	**19 (36)**	**5 (11)**	**4.41 (0.48**–**40.86), 0.19**	**21 (37)**	**5 (10)**	**3.01 (0.87**–**10.36), 0.08**
**Drug discontinuation**			**552 (1230)**	**284 (650)**	**2.01 (1.46**–**2.78),** < **0.001**	**795 (1614)**	**431(896)**	**1.79 (1.45**–**2.19),** < **0.001**

RevMan version 5.3 (The Nordic Cochrane Center, The Cochrane Collaboration; Copenhagen, Denmark) was used to conduct the primary analysis. Relative risk (RR) was determined for all studies using the Mantel-Haenszel random effects model with 95% confidence interval (CI) to establish the likelihood of adverse events. A secondary analysis was also performed to determine the RR for studies with follow up < 12 months and ≥12 months. Sensitivity analyses were used to show the robustness of the results. Heterogeneity was calculated using I^2^, a value which represents the percentage of variability in the effect risk estimate among studies due to heterogeneity rather than chance (I^2^ <25% considered as low, I^2^ between 25% and 75% as intermediate, I^2^ >75% considered as high). Begg's funnel plots method was utilized to investigate potential publication bias. A p-value of <0.05 was used to determine statistical significance.
